# Miscellaey

**Published:** 1912-10

**Authors:** 


### MISCELLAEY.

A story is being printed in some of our western exchanges, about a farmer who went to the city for a week's recreation and who became greatly interested in a melodrama. On the fourth consecutive evening that he had attended the play, the manager expressed his appreciation of his approval but advised him to go somewhere else and see something different. "Not on your life," replied the visitor. "You know the end of the second act where Don Juan Montague jumps out of the window just before the unexpected return of the husband? Well, some night, that cuss is going to get caught and I want to be there to see the fun."

Probably among the doctors who have laughed at this story—and at the Jay—are 57 who have been putting in from five to ten years hard work, doing the same show over and over again, expecting to be overtaken by a promotion that was just exactly as likely to do so as the injured husband in the melodrama was likely to catch the villain—and for exactly the same reason, that the whole thing had been carefully rehearsed.

